# Mechanical Anisotropy and Surface Roughness in Additively Manufactured Parts Fabricated by Stereolithography (SLA) Using Statistical Analysis

**DOI:** 10.3390/ma13112496

**Published:** 2020-05-30

**Authors:** Sunil Aravind Shanmugasundaram, Jafar Razmi, Md Jamal Mian, Leila Ladani

**Affiliations:** 1 Mechanical Engineering Department, Stanford University, Stanford, CA 94305, USA; sunilsvk@stanford.edu; 2 Ira A. Fulton Schools of Engineering, Arizona State University, Tempe, AZ 85281, USA; mmian2@asu.edu (M.J.M.); ladani@asu.edu (L.L.)

**Keywords:** additive manufacturing, stereolithography, tensile testing, anisotropy, surface roughness, aging effect, design of experiments, analysis of variance, Taguchi methods

## Abstract

In this study, the degree of mechanical anisotropy was investigated through tensile testing of specimens built in different orientations and designed according to the ASTM D638 standard. The mechanical properties that were evaluated include Young’s modulus, ultimate tensile strength (UTS), and percentage elongation. Additionally, physical properties, such as mean surface roughness (Ra), density and dimension of the cross-sectional area, were also measured. These properties were then compared with the available standard data to see how SLA performs comparing to the traditional manufacturing methods. The obtained modulus of elasticity and UTS values of the printed samples were 2481 ± 50 MPa and 51.9 ± 1.3 MPa respectively, which were very similar to the standard data (2550 and 52 MPa, respectively) as provided by the material suppliers. The percentage elongation values (4.8% ± 0.4%) were a bit lower than the expected value of 6%. However, the surfaces of all the printed samples were quite smooth, with a surface roughness range of 2.28 ± 0.59 µm. A design of experiments was created to study the influence of the independent variables such as build orientation and angular orientation on the mechanical properties. Extensive statistical analysis, using the Taguchi method and analysis of variance (ANOVA), was performed to examine the effect of these independent variables on the mechanical properties. The SLA printed parts can be classified as isotropic since the build orientation and the angular orientation did not have a statistically significant impact on the mechanical properties. The effect of aging on the mechanical properties was also evaluated and it shows that the specimens that had been aged for a longer time resulted in superior mechanical properties. For example, the UTS increased from 24 to 54 MPa when the sample aligned parallel to the XY plane was aged from 1 week to 6 months, respectively. This significant increase implies that aging has a substantial effect on the mechanical properties of the parts fabricated by stereolithography. The resin used for this study, Visijet Sl Clear, produced very consistent mechanical properties in different directions.

## 1. Introduction

Additive manufacturing (AM) is the current state-of-the-art in manufacturing and has revolutionized many industries. Key advantages of AM include significant economic benefits due to a reduction in the waste material produced during manufacturing, lower production time, elimination of complex tools, and lower energy cost and feasibility of complicated and advanced designs that are not feasible with traditional manufacturing [1,2,3]. Additive processes, as opposed to subtractive processes, involve the building of parts layer by layer [4,5,6]. The AM technique that is investigated in this study is stereolithography. The underlying principle of this technique is photopolymerization, wherein a liquid photopolymer resin gets converted to a solid polymer when exposed to ultraviolet laser radiation [7]. Analogous to other AM techniques, the final part is formed by successive layer addition [8]. Each layer of the resin material is cured, which refers to the process of hardening of material due to the crosslinking of polymer chains, and the curing process continues for the next layer [9,10]. The overlap of the cured layers results in the final part. This technique produces 3D objects with excellent surface finish, and very low stair-stepping effect, which makes it suitable in various biomedical and aerospace applications. For example, aeroelastic airfoils, cabin accessories, seatbacks, and entry doors are some aerospace products that can be produced using SLA [8]. Biodegradable and biocompatible polymers can be used for the production of scaffolds and many other medical applications like surgical tools, hearing aids, and dental appliances [10,11,12]. An intrinsic property of AM is mechanical anisotropy, which results in varied mechanical properties in different orientations since parts are built by layer addition [3]. This anisotropic behavior could be desirable or undesirable given design and application needs. The outcome of this project is to determine the level of mechanical anisotropy and evaluate the effectiveness of SLA for repeatability of manufacturing parts with as low anisotropy as possible. Having a lower anisotropy would ensure that properties of the manufactured product are uniform along different directions [2]. Consequently, the quality of products produced using SLA can be significantly improved by controlling parameters appropriately for the type of application, and this could also potentially lower the cost of production in the long run. As a result, SLA can grow and consequently be used in more industries and wider applications.

In addition to anisotropy, surface finish plays a very important role, especially in sensitive applications such as in the aerospace industry. Surface roughness is an important parameter that defines the wear of the part when the parts are dynamically loaded in contact with other parts. Furthermore, roughness determines the fatigue life of the parts under dynamic mechanical or thermo-mechanical cyclic load [13].

Anisotropy has been the subject of evaluation for additively manufactured parts for many years [1,2,3,14]. In particular, in the area of polymers, Dulieu-Barton and Fulton analyzed the influence of different experimental parameters such as environmental variations, post-cure time, and batch variations on the mechanical properties of specimens manufactured by SLA [14]. They found that if layers are transverse to the axis of loading, the highest values for tensile strength and Young’s modulus were achieved. Furthermore, cure time and controlling the humidity and temperature of the curing environment were found to be critical parameters. They observed that the anisotropy can be minimized to as much as 3% if the part is kept in a controlled environment and properties are not influenced by the post-cure time. Melchels et al. also examined the fundamentals of stereolithography, the unique features of this technique relative to other additive manufacturing techniques, and its numerous applications in the field of biomedical engineering [15].

Cuesta et al. analyzed the impact of different experimental parameters such as resin type, layer height, part orientation, and cleaning operations in the micro-SLA process, which refers to SLA for small scale production in the medical, dental, and jewelry fields [16]. They concluded that it was imperative to judiciously control the different printing parameters to improve the micro-SLA process. Lan et al. established the design criteria for suitable fabrication orientations using SLA, considering the surface quality, build time, and support structures [17]. They discovered that sloped surfaces manufactured by SLA produce parts with stepped surface texture, which can be reduced by orientating features either vertically or horizontally. They proposed two criteria to evaluate the surface quality. The first one maximized the area of non-stepped surfaces and the second one minimized the area of the worst quality.

Quintana et al. studied the influence of build orientation parameters on the mechanical properties of SLA manufactured parts, designed by ASTM D-638 type 1 configuration using a statistical design of experiments (DOE) [18]. Their DOE tested three independent factors which were axis, layout, and position. They observed that axis and position did not have a significant influence on the UTS or E values from a statistical analysis of the experimental data. However, the layout, either flat or edge, had a statistically significant impact on the UTS and E values, and the differences were around 3.53% for the UTS values and 4.59% for the E values. They concluded that the SLA parts cannot be considered as isotropic from a statistical viewpoint, although the differences appear to be negligible. Puebla et al. investigated the effect of aging, preconditioning, and build orientation on the mechanical properties of SLA manufactured parts, designed by ASTM D-638 type 1 configuration, using a design of experiments (DOE) and random tensile testing of the samples [19]. They observed that the samples that had been aged the least (4 days) and preconditioned based on ASTM recommended standards had the lowest values of UTS. Furthermore, the samples with an orientation of flat, wherein the layers were oriented along the thickness of the samples, recorded the lowest UTS and E values, and were statistically different from samples with orientations of edge or vertical. The samples with vertical orientation, wherein the layers were oriented along the length of the sample, generated the highest values of UTS and E. They concluded that conventionally manufactured SLA specimens cannot be classified as isotropic since the different orientations of the layers generated statistically different mechanical properties.

Saleh et al. evaluated the mechanical properties of two rapid prototyping (RP) techniques, stereolithography (SLA), and laser sintering (LS) [20]. They observed that SLA samples were isotropic by performing tensile, flexure, and impact tests for different build orientations of flat, edge, and upright. On the other hand, they discovered that LS samples were anisotropic. Hague et al. evaluated the mechanical properties of two state of the art stereolithography (SLA) resins for purposes of end-use parts, Accura SI40 and SL7560, and investigated the degree of anisotropy [21]. They observed that parts fabricated by both SL7560 and Accura SI40 can be classified as isotropic and therefore, different build orientations such as edge, flat, or upright, do not significantly impact mechanical properties. 

SLA, although in existence for the last couple of decades, is still relatively new and not sufficiently explored to fully comprehend the influence of experimental parameters such as angular orientations on the mechanical properties. Although the degree of mechanical anisotropy for SLA parts has been evaluated by other researchers, contradictory conclusions have been drawn [7,18,19,20,21]. In this project, a thorough effort was made to determine the level of mechanical anisotropy and evaluate the effectiveness of SLA for repeatability of manufacturing parts with as low anisotropy as possible. Since this manufacturing technique already possesses several key advantages over conventional methods, exploring how variations in experimental parameters impact the mechanical properties, can help in further improving products by tailoring experiments to get superior mechanical properties. The current project aims to remove any ambiguity and unequivocally establish the isotropic or anisotropic nature of SLA parts. Additionally, since adequate research has not been conducted on the influence of angular orientations around different principal axes on the mechanical properties, this current project strives to thoroughly investigate the influence of angular orientations on the mechanical properties of SLA parts through a thorough design of experiments, mechanical testing, and comprehensive statistical analysis. The influence of these different build parameters is extended beyond the standard mechanical properties such as UTS, E, and percentage elongation, to physical characteristics such as surface roughness.

## 2. Design of Experiments

**Figure 1:**
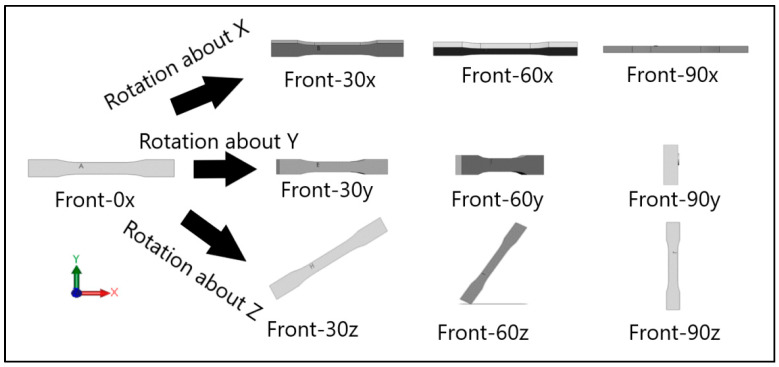
Front view of front-built samples, 0°, 30°, 60°, and 90° from left to right for each axis rotation (separate rows for x, y, and z-axis), respectively.

**Figure 2:**
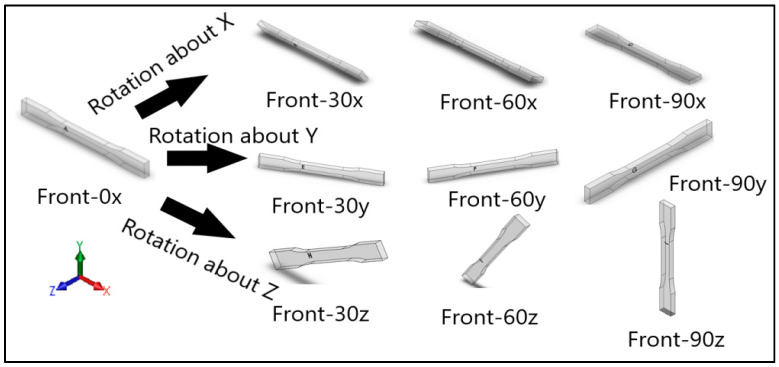
Isometric view of front-built samples, 0°, 30°, 60°, and 90° from left to right for each axis rotation (separate rows for x, y, and z-axis), respectively.

**Figure 3:**
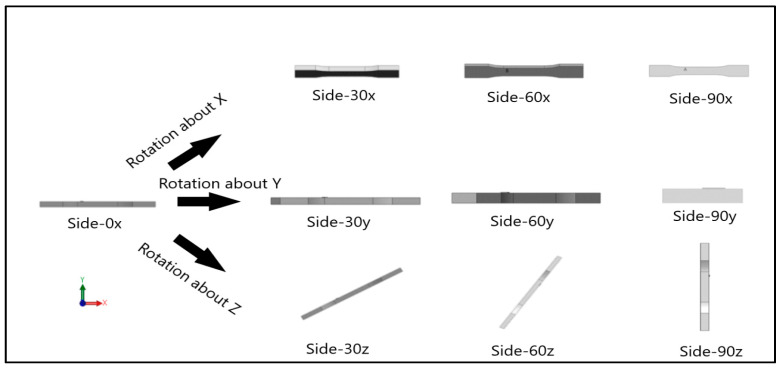
Front view of side-built samples, 0°, 30°, 60°, and 90° from left to right for each axis rotation (separate rows for x, y, and z-axis), respectively.

**Figure 4:**
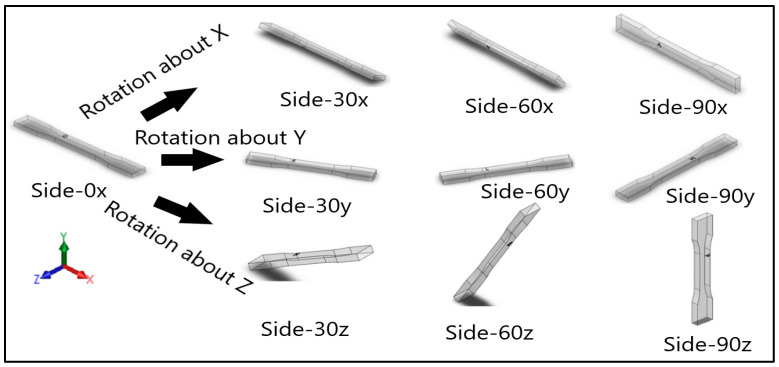
Isometric view of side-built samples, 0°, 30°, 60°, and 90° from left to right for each axis rotation (separate rows for x, y, and z-axis), respectively.

**Figure 5:**
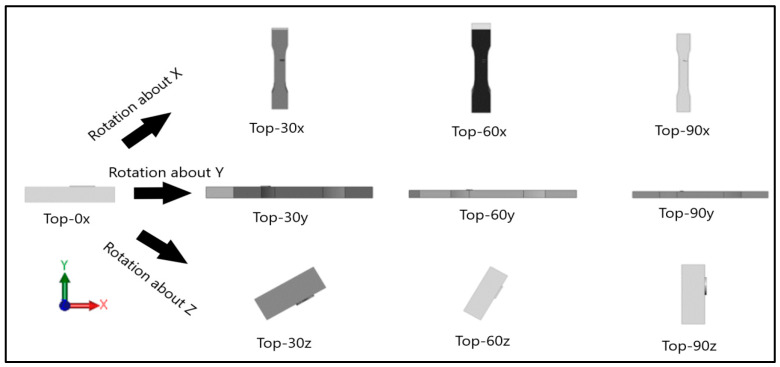
Front view of top-built samples, 0°, 30°, 60°, and 90° from left to right for each axis rotation (separate rows for x, y, and z-axis), respectively.

**Figure 6:**
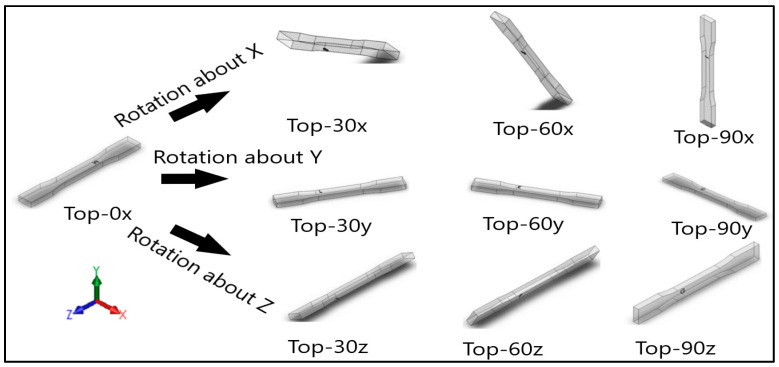
Isometric View of top-built samples, 0°, 30°, 60°, and 90° from left to right for each axis rotation (separate rows for x, y, and z-axis), respectively.

Dog-bone specimens were designed based on the ASTM D638-14 standard [22]. A design of experiment (DOE) was utilized to build samples under different build orientations, as shown in Table 1. The two factors that were assessed included the build orientation and the angular orientation. Samples were fabricated in three different build orientations: Front, Side, and Top. In the front samples, the layers are stacked through the thickness, while in the side samples, the layers are stacked along the width, and in the top samples, the layers are stacked in the longitudinal direction of the specimen. In addition to the different build orientation, the angular orientation of the samples was investigated by rotation of the samples by 30°, 60°, and 90° around all the three-principal axis, x, y, and z respectively in each build orientation. Since some combinations of the factors resulted in repeated configurations, it was necessary to eliminate the repeated combinations and only retain the unique ones. The DOE in Table 1 lists only the unique configurations and they have been labeled accordingly. The following figures show the CAD models for different build and angular orientations in different views such as the XY plane and isometric views. In each of these figures, the sample is rotated from a base configuration in increments of 30° from 0° to 90° around the three principal axes, respectively. Figure 1 and Figure 2 represent the different angular orientations for the front-built samples, while Figure 3 and Figure 4 represent side-built samples, and Figure 5 and Figure 6 depict top-built samples.

## 3. Sample Preparations

**Figure 7:**
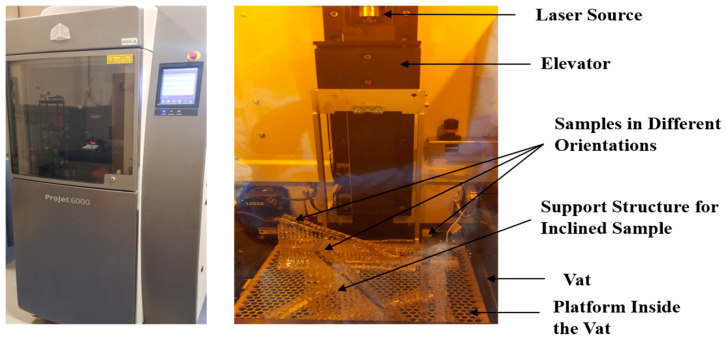
Outer view (left) and Inner view (right), with the printed samples of the used SLA 3D printer, ProJet 6000 HD.

All the finalized unique specimens were then printed using an SLA 3D printer, ProJet 6000 HD (3D Systems, Rock Hill, SC, USA), as shown in Figure 7. The resin used for this project was Visijet Sl Clear, also known as Accura ClearVue, which is a UV curable plastic that is crystal clear and certified USP Class VI (3D Systems, Rock Hill, SC, USA) [23]. The performance of parts fabricated by SLA depends on the quality of the resin material used. Different resin materials have trade-offs with mechanical properties and desired physical characteristics. In comparison with other conventional resin materials for this printer, such as Visijet Sl Tough and Visijet Sl Flex, Visijet Sl Clear has higher tensile strength and tensile modulus. Visijet Sl Clear was chosen for this project as it has better tensile properties rather than emphasizing other parameters such as impact or flexural performances. Since it is certified USP Class VI, Visijet Sl Clear is suitable for applications in the medical industry such as dental products and hearing aids. The manufacturer provided specifications for the mechanical properties obtained using this resin include UTS of 52 MPa, E of 2560 MPa, and elongation at break of 6%. The experimental data that were obtained from the tensile tests of the different configurations were compared with these standard data to examine the level of repeatability of the experiments and the degree of anisotropy. The physical appearance of this resin is transparent and polycarbonate-like. Visijet Sl Clear has high stiffness, durability, tensile strength, and impact strength, which enables this resin to compete with traditional plastics. The settings used for printing the samples included Ultra High Definition (UHD) with a layer resolution of 100 μm, a net build volume of 254 mm× 254 mm × 264.16 mm, and a resolution equivalent to 4000 DPI [24]. Given the dimensions of the build volume and that of the samples, it was possible to print only 10 samples in one print job. Including the supports, each print job required 176.38 mL of Visijet Sl Clear and 14 h to print. After the samples were printed, the support structures were removed manually. The samples were then subjected to cleaning using isopropyl alcohol (IPA) in two baths. During the first bath, the samples were soaked with IPA and agitated with compressed air at 100 psi. The process was repeated during the second bath. After the cleaning process, the samples were kept in a curing chamber, ProCure 350 UV Chamber (3D Systems, Rock Hill, SC, USA), and cured in 405 nm UV light at room temperature for around 20 min [25]. To test the repeatability of the experiment and get better accuracy, six batches of these twenty configurations were manufactured to get a total of one hundred and twenty samples.

To check the dimensional accuracy of the samples, after the build, the width and thickness of one batch of samples were measured and recorded using a Mitutoyo 500-196-30 AOS Digital Vernier Caliper (Aurora, IL, USA). The dimensions of the actual samples were within two percent of the specified dimensions of the CAD models. Additionally, the density of each of the samples was measured, and it was within four percent of the specified solid density of 1.17 g/cm^3^ of the resin used for this analysis.

## 4. Experimentation

### 4.1. Surface Roughness Measurement

The surface profile of a part can be measured by instruments such as profilometers, which create 2D graphs of the shape of the surface that is sectioned by the plane. Contact methods to record the surface profile generally involve a stylus. The stylus records the profile by vertical displacement dependent on position. As mentioned before, the roughness can be classified as the shortest wavelength deviations of the surface from the intended surface. This profile is attained by applying a filter to attenuate the longer wavelengths that are part of waviness and form error. A minimum cutoff can be set as part of a high pass filter, to exclude the very shortest wavelengths that may be noise. The roughness of a part is an important parameter that determines the friction of a part in contact with another surface. For parts that are intended to be moving or where there are dynamics involved, the roughness plays an important role in determining the wear of the parts [26]. Furthermore, it has been shown that a crack typically starts at the surface of the parts and larger surface asperities could cause shorter fatigue life when parts experience cyclic mechanical or thermomechanical loading.

While several parameters can quantify surface roughness, the most important parameter is the average surface roughness, Ra. The Ra refers to the absolute value of the roughness, around the mean line, integrated over the evaluation length of the sample, as represented in Equation (1).

(1) $$R_{a}=\frac{1}{L}\int_{0}^{L}\left|z\left(x\right)\right|dx$$

**Figure 8:**
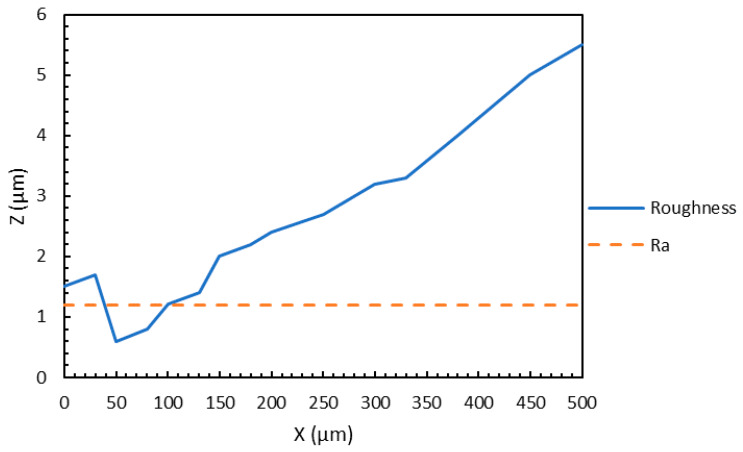
Sample measurement for surface roughness.

The different terms used in Equation (1) are $R_{a}$, which denotes the mean surface roughness; L, which refers to the total evaluation length; and z(x), which is the height of the profile along the x-direction. In this study, the average surface roughness (Ra) of the samples at different orientations was measured using a 3D optical profilometer named MicroXAM-100 Digital Interferometer (KLA, Milpitas, CA, USA). After calibrating the instrument by adjusting instrument parameters such as the objective and Z range, the roughness measurements were recorded at different areas of the surfaces for each sample. Readings were taken for two surfaces, one along the thickness of the sample and the other along the gage section of the sample. The average surface roughness was measured from the optical images, taken from random areas of the surfaces, using the optical profilometer. Although Ra values for both surfaces were recorded, only the higher Ra value was used for further analysis. According to the initial hypothesis, the higher Ra value will be a better indicator of the surface roughness of the sample and more beneficial to understand the implications of surface roughness. For each of the 20 samples, six measurements were taken to get a more accurate mean Ra value. Figure 8 is a representative of one such measurement. In this case, the interferometer was used to take a measurement along the gage section of Sample A. The Ra value, as seen from the figure, is 1.2 μm for this case.

**Figure 9:**
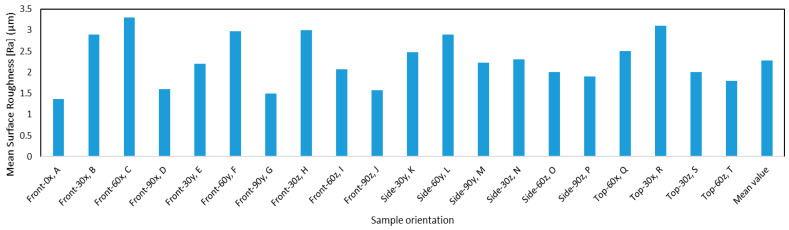
The mean surface roughness for the different orientations.

The mean surface roughness for each of the different configurations was then plotted in the form of a bar chart, as seen in Figure 9. Furthermore, higher roughness values were expected in the direction parallel to the build direction. The data that were acquired were consistent with this hypothesis. It can also be seen the maximum roughness occurs for sample C, which has an orientation of 60° rotations around the x-axis and layers stacked along the thickness. Although Figure 9 offers a visual representation of the average surface roughness of the different orientations, the significance of angular and build orientations on the roughness cannot be established qualitatively. Therefore, in a later section, a statistical analysis will be performed to qualitatively determine the impact of build parameters on the surface roughness.

### 4.2. Mechanical Testing

**Figure 10:**
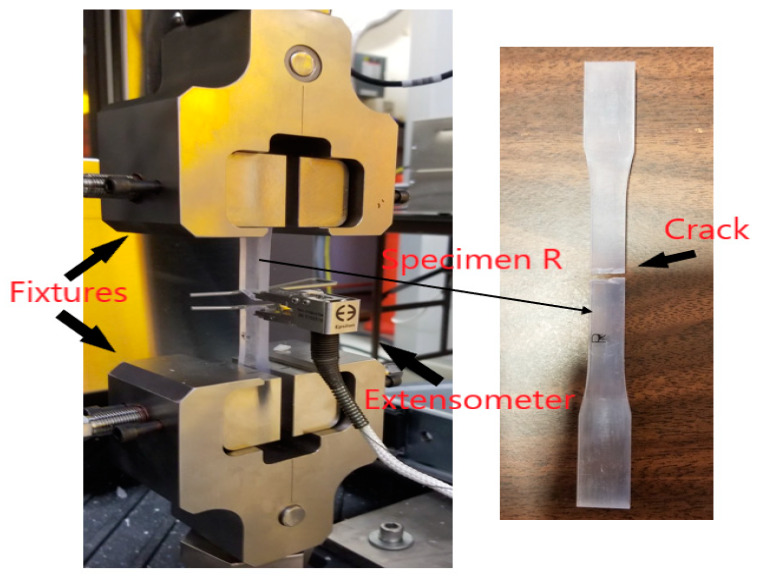
The axial extensometer engaged with a test specimen.

The tensile tests of the 120 dog bone specimens were completed in random order using an MTS Criterion^®^ Series 40 Electromechanical Universal Test System (Eden Prairie, MN, USA) containing an MTS C45.504 extended length load frame. The test system had a load cell modeled as MTS LPS.504 with a force capacity of 50 kN (11 kip). All the tests were carried out in strain control mode, with the strain rate of 0.5 mm/mm/min following the ASTM D638-14 standard [22]. The strain data were collected using an Epsilon axial extensometer modeled as 7642-010M-075M, with a gage length of 10 mm (Jackson, WY, USA). Six specimens of each build orientation were tested using this test system. The average data acquired from the successful tensile tests for all the configurations have been shown in Table 2. The mechanical properties that have been recorded include modulus of elasticity (E), ultimate tensile strength (UTS), stress at break, strain at break, and percentage elongation. The average surface roughness values are also presented for comparison. In the table, the last row provides the standard values of E, UTS, and percent elongation specified by the SLA printer provider for VisiJet SL Clear material. Figure 10 represents the experimental setup for one of the tensile tests and includes the different components such as fixtures, extensometer, the sample, and results after the test.

**Figure 11:**
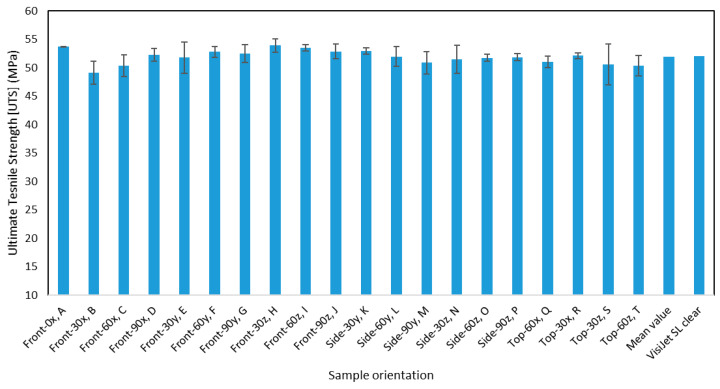
The ultimate tensile strength for the different orientations.

The mechanical properties E, UTS, and percentage elongation were also plotted in the form of bar charts, as seen in the following figures, to visually understand the effect of changing the build orientation on these properties. From these graphs, it can be observed that there is very little variation in these properties due to changes in the build orientations. The mean value for each of the mechanical properties was found to be very close to the standard values of VisiJet SL Clear material. Error bars, which were found by computing the standard error from the standard deviation, are displayed for each measurement to understand the level of confidence and variation for the different measurements. Some measurements had smaller error bars because of the high level of repeatability of the experiments that generated consistent results. While in other cases, some types of errors such as human error involved in calibrating the fixtures for the tensile test resulted in higher standard error. In Figure 11, the ultimate tensile strength for the different orientations is plotted along with the error bars. From here, the initial hypothesis that can be formulated is that the different orientations appear to have a small impact on the ultimate tensile strength. This initial hypothesis is validated by the statistical analysis that is performed in a later section. The mean value of the ultimate strength value for all the orientations is very similar to the value provided by the manufacturer.

**Figure 12:**
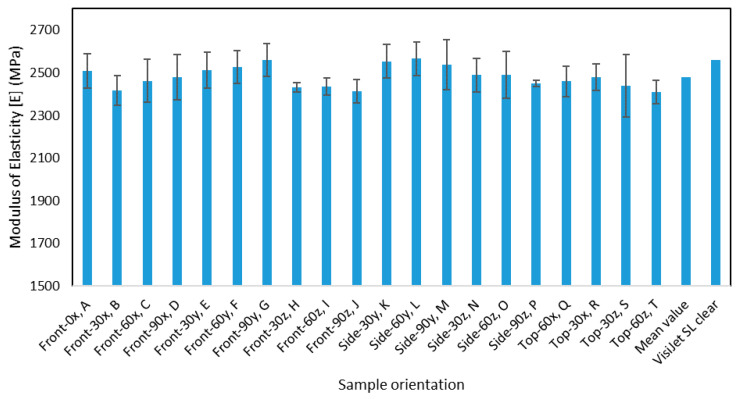
The modulus of elasticity for the different orientations.

Figure 12 depicts the modulus of elasticity as a function of the different angular and build orientations. It can be observed that there is some variation in the modulus of elasticity, and it is not as consistent as the previous mechanical property. Although the modulus of elasticity is an intrinsic property that depends on the intermolecular bonding strength, the variation can be attributed to the aging of the different samples more than the impact of the build orientations. The effect of aging on the mechanical properties is discussed in a later section.

**Figure 13:**
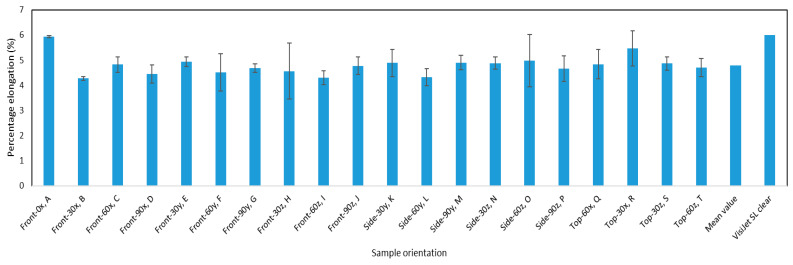
The percentage elongation for the different orientations.

Figure 13 represents the bar chart of the percentage elongation of the different samples plotted against the build orientations. Based on the initial hypothesis, the percentage elongation appears to be fairly consistent with the different orientations. This hypothesis is tested by the statistical analysis that is detailed in the next section.

## 5. Data Analysis and Discussion

Although the graphs represent visual representation of the data, they do not offer much insight into the significance of the various measurements. Since the objective of this project is to determine the level of anisotropy that exists in the parts manufactured by SLA, it is necessary to qualitatively analyze the measurements taken for the different orientations. The analysis of variance (ANOVA) method was performed to investigate the influence of the different independent variables on the different response variables [27,28,29]. The fundamental principle behind ANOVA is hypothesis testing. By comparing the dependent variable’s means at each of the different factor levels, ANOVA can determine the significance of the factors. Similar to other statistical tests that use hypothesis testing, ANOVA tests both the null and the alternate hypothesis. The means of all the factor levels are the same in the null hypothesis, while at least one factor level’s mean is different in the alternate hypothesis. As the name implies, ANOVA uses variances to check if the means are different. ANOVA will determine whether one can reject the null hypothesis, which implies that one accepts the alternate hypothesis. Rejecting the null hypothesis implies that the independent variable is significant and that it influences the dependent variable statistically. Given the combination of the factors and elimination of duplicates, the Taguchi design of experiments, which employs a fractionated orthogonal array [30], is used to conduct ANOVA. Since the Taguchi method uses an orthogonal array, the resulting array is balanced, since the factor levels are weighted equally. Therefore, the Taguchi DOE allows the main effects to be investigated independently of the interaction effects. The term main effect implies that there is an impact of different levels of a factor on the response variable. In this method, the analysis was set up such that there are three independent variables with three levels or groups each. If the design of experiments was set up in this manner, then it would require only nine runs to analyze the influence of the different factors on the response variable. The three independent variables are build orientation, axis, and angular orientation. Build orientation refers to the way the layers are arranged. Consequently, there are three levels for this factor: front, side, and top. As mentioned previously, in the front samples, the layers are stacked along the thickness, while in the side samples, the layers are stacked along the width, and in the top samples, the layers are stacked in the longitudinal direction of the samples. The other two factors represent the rotation of the samples by 30°, 60°, and 90° around all the three principal axes, x, y, and z, respectively. The design of experiments through this method is set up in Table 3. The dependent variables investigated are mechanical properties such as UTS, E, percent elongation, and Ra, respectively. The first three columns of Table 3 represent the various levels of the independent variables and the next four columns represent the response variables corresponding to the different factor levels. Before performing the ANOVA, it is necessary to state the null and alternate hypotheses. The null hypothesis that is suitable for this DOE would state that the means of the respective mechanical properties are the same for different levels of the respective independent variables. The alternate hypothesis, as the name implies, would state the means of the respective mechanical properties are not the same for different levels of the respective independent variables.

The results obtained through the Taguchi analysis include ANOVA for the mechanical properties. The different terms used in Table 4 include SS, which refers to the sum of squares; df, which refers to degrees of freedom; and F, which is the F ratio (ratio of means between groups and within groups). Between-group variability denotes the variation of the means of different groups while within-group variability refers to the variation within a group that is considered independently of the other groups. The key result from ANOVA is the ratio of between-group variance and within-group-variance that is referred to as the F ratio. If the between-group variance is much higher than the within-group variance, the means are not equal. The F ratio that is calculated is compared with a term denoted as the F critical. F critical refers to the value of the F statistic from the F distribution corresponding to the confidence level specified. If the F ratio is higher than F critical, then the data are significant. The other term that can be used to determine statistical significance is the *p*-value. The *p*-value is the probability that the results obtained are as extreme as the sample data, assuming the validity of the null hypothesis. The *p*-value is compared with the alpha value to determine if the null hypothesis can be rejected. If the *p*-value is lower than the alpha value, then the data are significant. The alpha value is taken as 0.05 as that is the standard value used in most applications and refers to a 95% confidence. As seen in Table 4, the *p*-value for all three independent variables is greater than the alpha value. This implies that the null hypothesis for each of the independent variables cannot be rejected. Therefore, changing the three independent variables does not have a statistically significant impact on the UTS.

As seen in Table 5, the *p*-value for the build orientation and angular orientation is greater than the alpha value. This implies that the null hypothesis for these independent variables cannot be rejected. However, since the *p*-value is lower than alpha for axis, the null hypothesis for the axis can be rejected. Therefore, changing the build orientation or angular orientation does not have a statistically significant impact on the E but changing the axis does statistically influence the E.

As seen in Table 6, the *p*-value for all three independent variables is greater than the alpha value. This implies that the null hypothesis for each of the independent variables cannot be rejected. Therefore, changing the three independent variables does not have a statistically significant impact on the percentage elongation.

**Figure 14:**
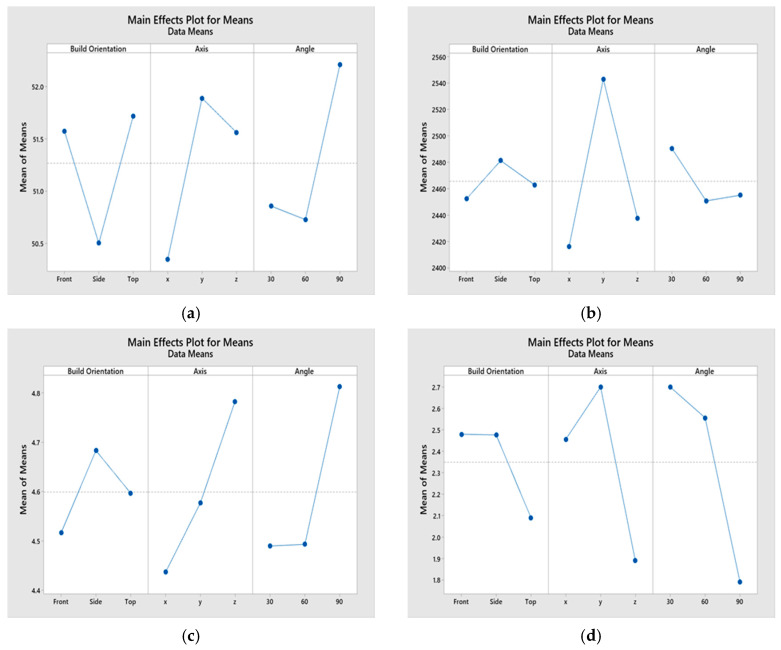
Main effects plot for (**a**) UTS, (**b**) modulus of elasticity, E, (**c**) percent elongation, and (**d**) surface roughness, Ra using Taguchi analysis.

**Figure 15:**
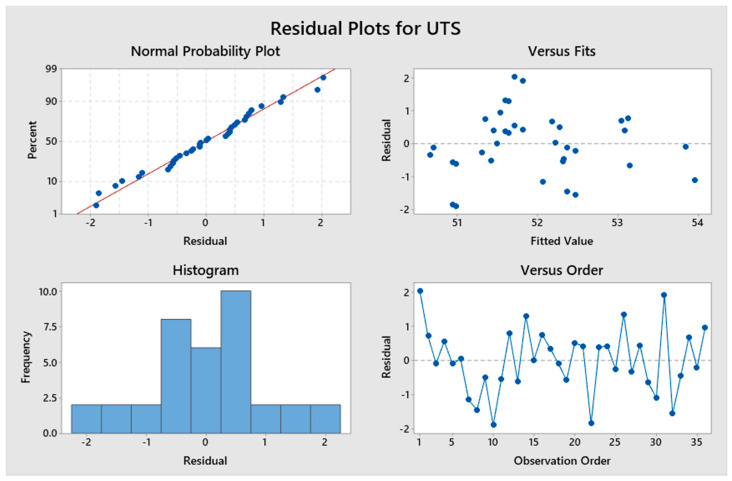
Residual plots for UTS through the two-way ANOVA method.

As seen in Table 7, the *p*-value for the build orientation is greater than the alpha value. This implies that the null hypothesis for this independent variable cannot be rejected. However, since the *p*-value is lower than alpha for both the axis and angular orientation, the null hypothesis for them can be rejected. Therefore, changing the build orientation does not have a statistically significant impact on the Ra, but changing the axis or angle does statistically influence the Ra. The results were then graphed in the form of main effects plots, as seen in Figure 14, to visually understand the impact of changing each of the three main factors on the response. The main effects plot should be interpreted by the slope of the line such that a steeper slope indicates a higher main effect of the factor. For example, in Figure 14a, when the level of the axis factor is changed from x to y, the resulting UTS is greatly impacted, as indicated by the high slope of the main effects line. Conversely, from Figure 14c, it can be observed that changing the angular orientation from 30° to 60° does not have an impact on the percentage elongation, as indicated by the nearly horizontal line. These graphs validate the ANOVA results obtained through the Taguchi analysis. The last component of the statistical analysis was the residual mean plots, as seen in Figure 15, which indicate the statistical adequacy of the model. Even though all the residual plots are not shown here because of space constraints, they verified that the chosen statistical models for the different mechanical properties were adequate. For example, the normal probability plots for UTS had data points very close to the straight line, which implies the model fits the data well.

**Figure 16:**
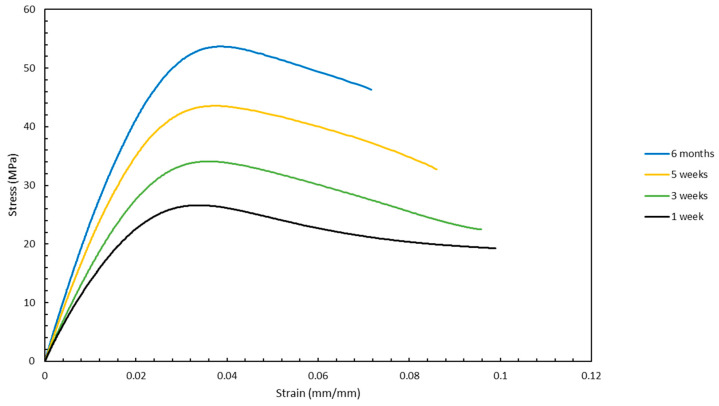
Variation of stress vs. strain curves with aging for samples front-0x, A.

**Figure 17:**
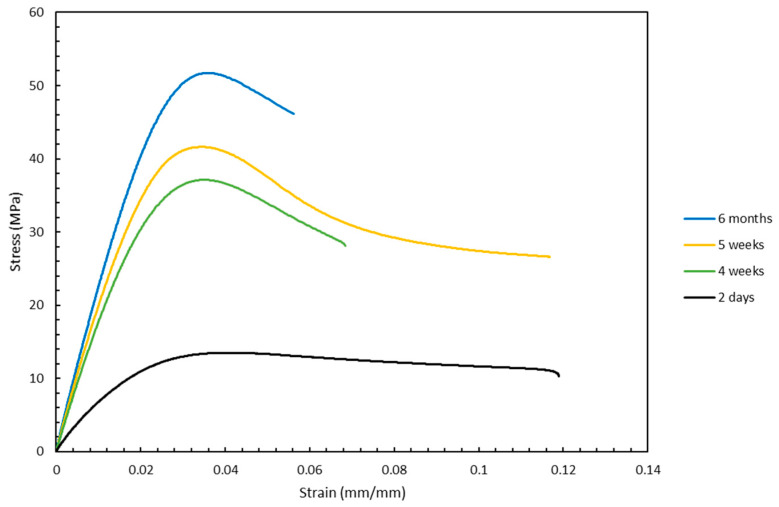
Variation of stress vs. strain curves with aging for sample front-60z, O.

Finally, another factor that was tested besides the orientations was the influence of the aging duration of the samples on the mechanical properties. As seen from the stress vs. strain curves in Figure 16 and Figure 17, the mechanical properties for the samples that were aged the most were superior to those that were aged the least. As seen in Figure 16, the UTS increased from approximately 24 to 54 MPa when the sample with an orientation of front-0x was aged from one week to six months. This indicates that aging has a significant influence on the mechanical properties of samples fabricated by stereolithography. Although these two figures show the effect of aging on mechanical properties only for front built samples that were rotated 0° around the x-axis and 60° around the z-axis, this trend is representative of the data that were collected for the other orientations as well.

The Taguchi design of experiments was derived based on the orthogonal array that resulted in fewest trials based on the given combinations of the factors. In the Taguchi method, three independent factors with three levels each and their impact on the response variables were investigated. Although the main effects plot and *p*-values determined that the different independent variables did not have a statistical impact on the mechanical properties, the effect of interaction between the different independent variables was not accounted for in the Taguchi method. Moreover, since the Taguchi design of experiments is highly fractionated, the interaction effect that can be determined through statistical analysis can be misleading. Therefore, to investigate the impact of one independent factor at a time on the response variable, another statistical method called one-way ANOVA was employed. One-way ANOVA is a statistical method where the influence of one independent variable with different levels on one dependent variable can be determined. Since each factor is analyzed independently, the interaction effect need not be considered an error. Based on the levels of a factor, the design of experiments setup for the one-way ANOVA will be different. The two independent variables that are investigated are the build orientations and angular orientations. The design of experiments setup and the ANOVA procedure are similar for the different dependent variables that are investigated such as UTS, E, percentage elongation, and Ra. For each of the dependent variables, the ANOVA results of the *p*-value, and confidence intervals established through the pooled standard deviation are presented. Table 8 represents the DOE setup for analyzing the dependent variable of E as a function of the independent variable of angular orientations. Each column entry represents the E for the different angular orientations.

Similar to the Taguchi DOE, the null hypothesis for this DOE would state that the means of the response variable, E, are the same for different levels of the angular orientation. The ANOVA results obtained are shown in Table 9, where the *p*-value of 0.618 is higher than the alpha value, which implies that the null hypothesis cannot be rejected. Therefore, angular orientation does not have a statistical impact on the modulus of elasticity.

Table 10 represents the number of observations, mean, standard deviation, and 95% confidence interval for E for each level of the angular orientation.

Table 11 represents the DOE setup for analyzing the dependent variable of E as a function of the independent variable of build orientations. Each column entry represents the E for the different build orientations.

The ANOVA results obtained are shown in Table 12, where the *p*-value of 0.683 is higher than the alpha value, which implies that the null hypothesis cannot be rejected. Therefore, build orientation does not have a statistical impact on the modulus of elasticity.

Table 13 represents the number of observations, mean, standard deviation, and 95% confidence interval for E for each level of the build orientation.

Table 14 represents the DOE setup for analyzing the dependent variable of UTS as a function of the independent variable of angular orientations. Each column entry represents the UTS for the angular orientations.

The ANOVA results obtained are shown in Table 15. As seen in Table 15, the *p*-value of 0.305 is higher than the alpha value, which implies that the null hypothesis cannot be rejected. Therefore, angular orientation does not have a statistical impact on the ultimate tensile strength.

Table 16 represents the number of observations, mean, standard deviation, and 95% confidence interval for UTS for each level of the angular orientation.

Table 17 represents the DOE setup for analyzing the dependent variable of UTS as a function of the independent variable of build orientations. Each column entry represents the UTS for the build orientations.

The ANOVA results obtained are shown in Table 18. As seen in Table 18, the *p*-value of 0.162 is higher than the alpha value, which implies that the null hypothesis cannot be rejected. Therefore, build orientation does not have a statistical impact on the ultimate tensile strength.

Table 19 represents the number of observations, mean, standard deviation, and 95% confidence interval for UTS for each level of the build orientation.

Table 20 represents the DOE setup for analyzing the dependent variable of percentage elongation as a function of the independent variable of angular orientations. Each column entry represents the percentage elongation for the angular orientations.

The ANOVA results obtained are shown in Table 21. As seen in Table 21, the *p*-value of 0.199 is higher than the alpha value, which implies that the null hypothesis cannot be rejected. Therefore, angular orientation does not have a statistical impact on the percentage elongation.

Table 22 represents the number of observations, mean, standard deviation, and 95% confidence interval for percentage elongation for each level of the angular orientation.

Table 23 represents the DOE setup for analyzing the dependent variable of percentage elongation as a function of the independent variable of build orientations. Each column entry represents the percentage elongation for the build orientations.

The ANOVA results obtained are shown in Table 24. As seen in Table 24, the *p*-value of 0.665 is higher than the alpha value, which implies that the null hypothesis cannot be rejected. Therefore, build orientation does not have a statistical impact on the percentage elongation.

Table 25 represents the number of observations, mean, standard deviation, and 95% confidence interval for the percentage elongation for each level of the build orientation.

Table 26 represents the DOE setup for analyzing the dependent variable of surface roughness as a function of the independent variable of build orientations. Each column entry represents the mean surface roughness for the build orientations.

The ANOVA results obtained are shown in Table 27. As seen in Table 27, the *p*-value of 0.942 is higher than the alpha value, which implies that the null hypothesis cannot be rejected. Therefore, build orientation does not have a statistical impact on the surface roughness.

Table 28 represents the number of observations, mean, standard deviation, and 95% confidence interval for Ra for each level of the build orientation.

Table 29 represents the DOE setup for analyzing the dependent variable of surface roughness as a function of the independent variable of angular orientations. Each column entry represents the mean surface roughness for the angular orientations.

The ANOVA results obtained are shown in Table 30. As seen in Table 30, the *p*-value of 0.000 is lower than the alpha value, which implies that the null hypothesis can be rejected. Therefore, build orientation does have a statistical impact on the surface roughness. Although a statistical significance was observed between the angular orientation and the surface roughness, it may occur due to the staircase effect, since layers that are parallel to the build direction were observed to have higher roughness. The surface where layers are parallel to the build direction is seen to have higher roughness compared to the surface with layers perpendicular to the build direction. The reason behind this increased surface roughness might be due to the staircase effect, with the angular orientation of the samples on the build table. However, from a qualitative perspective, the obtained surface roughness values are insignificant in terms of the quality of SLA printed parts as opposed to the other AM polymer parts.

Table 31 represents the number of observations, mean, standard deviation, and 95% confidence interval for Ra for each level of the angular orientation. 

The difference in the results obtained through the one-way ANOVA from the previous Taguchi methods results is that in the Taguchi methods, the variance considers three independent variables and uses a highly fractionated array instead of a full factorial one. However, the results that are obtained through both methods are the same and they validate each other. Taguchi DOE was selected due to the efficient setup with the fewest possible trials considering the different levels of the independent variables. To consider the possibility of interaction effects, one-way ANOVA was also performed. The results from both the statistical methods are similar and validate that the independent variables of build and angular orientations do not have a statistical impact on the different mechanical properties. Although some variation may be seen in the bar charts, the variation is not statistically significant and occurs due to possible sources of error such as human error involved in calibrating the test experiment and a difference in the aging of the samples. The level of mechanical anisotropy in SLA printed parts was investigated using two statistical methods. The one-way ANOVA and the Taguchi analysis both yielded similar results for all the different mechanical properties such as UTS, E, and percentage elongation. Since a large number of specimens were tested in this study, the one-way ANOVA is more comprehensive than the Taguchi analysis to investigate the anisotropy of these properties. The one-way ANOVA used a full factorial DOE to analyze the variance and determine the statistical significance of the independent variables on the dependent variables. A full factorial DOE is a comprehensive setup to analyze both the main effects and interaction effects of the independent variables. The one-way ANOVA confirmed that changing the build orientation or the angular orientation does not have a statistically significant influence on the UTS, E, and percentage elongation. These results were then substantiated through the Taguchi analysis and the main effects plots. Since the results obtained by the Taguchi method were similar and validated the results obtained by one-way ANOVA, the Taguchi method is more efficient due to the minimum number of trials.

## 6. Conclusions

Although the level of mechanical anisotropy for SLA parts has been investigated by several researchers, conflicting conclusions have been made. The current project has unambiguously established that SLA printed parts can be classified as isotropic. This indicates that aligning the part at different orientations around the build platform will result in similar properties along different directions. This allows SLA to have a tremendous advantage over other AM techniques such as FDM, which has been experimentally tested to produce anisotropic parts. Another key finding was the impact of the aging of the samples on the mechanical properties. It was discovered that aging had a direct relationship with the mechanical properties. This implied that the samples that had been aged for a longer period had far superior mechanical properties than inadequately aged samples and this result was consistent with other researchers. Lastly, the resin used for the SLA printed parts, Visijet Sl Clear, generated both consistent mechanical properties along different directions and good properties that are similar or superior to traditional plastics. This indicates that this process can be used in the biomedical and aerospace industries for producing lightweight and transparent end-user parts that require a good deal of mechanical strength.

## Figures and Tables

**Table 1:** Design of experiments.

Specimen No.	Build Orientation	Letters
1	Front–0x	A
2	Front-30x	B
3	Front-60x	C
4	Front-90x	D
5	Front-30y	E
6	Front-60y	F
7	Front-90y	G
8	Front-30z	H
9	Front-60z	I
10	Front-90z	J
11	Side-30y	K
12	Side-60y	L
13	Side-90y	M
14	Side-30z	N
15	Side-60z	O
16	Side-90z	*p*
17	Top-60x	Q
18	Top-30x	R
19	Top-30z	S
20	Top-60z	T

**Table 2:** Tensile test average results and mean surface roughness for all unique configurations.

Build Orientations	Modulus of Elasticity, E (Mpa)	Ultimate Tensile Strength (Mpa)	Percent Elongation (%)	Roughness (μm)	Coordinate System (Isometric)	
Front- 0x	2508.57	53.75	5.940	1.37	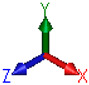	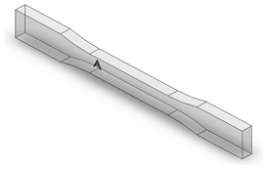
Standard Error	81.2782	0.0316	0.0443			
Front-30x	2417.25	49.09	4.270	2.90	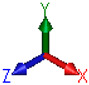	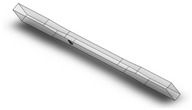
Standard error	68.1542	2.0445	0.0759			
Front-60x	2462.01	50.37	4.820	3.30	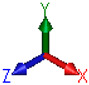	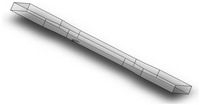
Standard error	101.0281	1.8789	0.3137			
Front-90x	2479.51	52.26	4.450	1.60	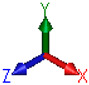	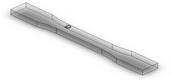
Standard error	106.0512	1.1614	0.3599			
Front-30y	2513.16	51.77	4.940	2.20	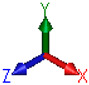	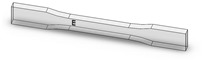
Standard error	83.6156	2.7073	0.1861			
Front-60y	2526.14	52.78	4.510	2.97	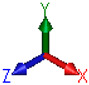	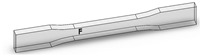
Standard error	76.2510	0.9993	0.7415			
Front-90y	2559.82	52.50	4.680	1.50	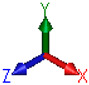	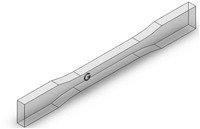
Standard error	77.4150	1.6079	0.1747			
Front-30z	2430.41	53.92	4.560	3.00	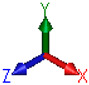	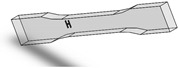
Standard error	21.6057	1.2071	1.1074			
Front-60z	2434.02	53.50	4.290	2.07	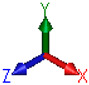	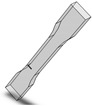
Standard error	39.8991	0.5710	0.2758			
Front-90z	2413.92	52.86	4.770	1.57	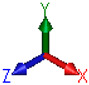	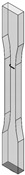
Standard error	55.3691	1.2657	0.3471			
Side-30y	2553.24	52.94	4.890	2.47	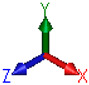	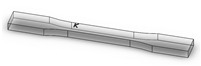
Standard error	78.6780	0.5482	0.5371			
Side-60y	2565.51	51.98	4.320	2.90	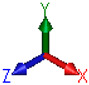	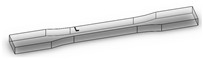
Standard error	77.7440	1.7800	0.3329			
Side-90y	2537.71	50.91	4.900	2.23	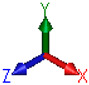	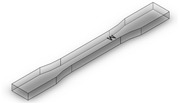
Standard error	116.4822	1.9725	0.2902			
Side-30z	2489.12	51.51	4.880	2.30	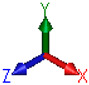	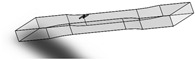
Standard error	78.2868	2.4841	0.2437			
Side-60z	2490.35	51.73	4.980	2.00	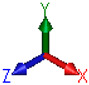	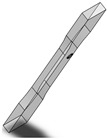
Standard error	108.6213	0.6039	1.0423			
Side-90z	2449.75	51.87	4.660	1.90	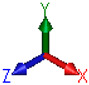	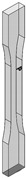
Standard error	13.2113	0.6470	0.5158			
Top-60x	2459.33	51.05	4.840	2.50	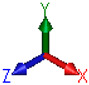	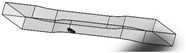
Standard error	72.9703	0.9774	0.5860			
Top-30x	2477.90	52.10	5.470	3.10	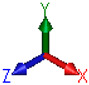	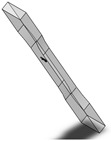
Standard error	62.3721	0.5079	0.6978			
Top-30z	2438.71	50.60	4.870	2.00	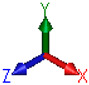	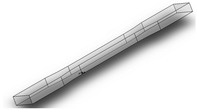
Standard error	145.9098	3.6019	0.2659			
Top-60z	2409.25	50.32	4.700	1.80	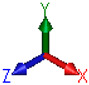	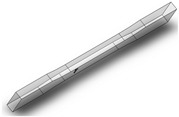
Standard error	55.1534	1.8159	0.3654			
VisiJet SL Clear	2560	52	6			

**Table 3:** Taguchi orthogonal array setup.

Build Orientation	Axis	Angle (°)	UTS (MPa)	E (MPa)	Percentage Elongation (%)	Ra (μm)
Front	x	30	49.09	2417.25	4.27	2.90
Front	y	60	52.78	2526.14	4.51	2.97
Front	z	90	52.86	2413.92	4.77	1.57
Side	x	60	49.09	2417.25	4.27	2.90
Side	y	90	50.91	2537.71	4.90	2.23
Side	z	30	51.51	2489.12	4.88	2.30
Top	x	90	52.86	2413.92	4.77	1.57
Top	y	30	51.98	2565.51	4.32	2.90
Top	z	60	50.32	2409.25	4.70	1.80

**Table 4:** ANOVA for UTS obtained through Taguchi analysis.

Source	DF	Seq SS	Adj SS	Adj MS	F	*p*
Build Orientation	2	2.653	2.653	1.326	0.34	0.746
Axis	2	3.969	3.969	1.984	0.51	0.662
Angle	2	4.030	4.030	2.015	0.52	0.659
Residual Error	2	7.783	7.783	3.892		
Total	8	18.435				

**Table 5:** ANOVA for E obtained through Taguchi analysis.

Source	DF	Seq SS	Adj SS	Adj MS	F	*p*
Build Orientation	2	1286.9	1286.9	643.5	1.83	0.353
Axis	2	27,747.6	27,747.6	13,873.8	39.44	0.025
Angle	2	2854.5	2854.5	1427.3	4.06	0.198
Residual Error	2	703.5	703.5	351.8		
Total	8	32,592.5				

**Table 6:** ANOVA for percentage elongation obtained through Taguchi analysis.

Source	DF	Seq SS	Adj SS	Adj MS	F	*p*
Build Orientation	2	0.04169	0.04169	0.02084	0.38	0.724
Axis	2	0.18249	0.18249	0.09124	1.67	0.375
Angle	2	0.20696	0.20696	0.10348	1.89	0.346
Residual Error	2	0.10936	0.10936	0.05468		
Total	8	0.54049				

**Table 7:** ANOVA for Ra obtained through Taguchi analysis.

Source	DF	Seq SS	Adj SS	Adj MS	F	*p*
Build Orientation	2	0.30162	0.30162	0.15081	5.62	0.151
Axis	2	1.03642	1.03642	0.51821	19.33	0.049
Angle	2	1.43642	1.43642	0.71821	26.79	0.036
Residual Error	2	0.05362	0.05362	0.02681		
Total	8	2.82809				

**Table 8:** DOE for the impact of angular orientations on E.

0 Degrees	30 Degrees	60 Degrees	90 Degrees
2508.57	2417.25	2462.01	2479.51
2508.57	2513.16	2526.14	2559.82
2508.57	2430.41	2434.02	2413.92
2479.51	2553.24	2565.51	2537.71
2479.51	2489.12	2490.35	2449.75
2479.51	2477.90	2459.33	2508.57
2537.71	2438.71	2409.25	2479.51
2537.71	2462.01	2417.25	2413.92
2537.71	2565.51	2553.24	2559.82

**Table 9:** ANOVA results for E as a function of angular orientations.

Source	DF	Adj SS	Adj MS	F-Value	*p*-Value
Angular orientation	3	4514	1505	0.60	0.618
Error	32	79,787	2493		
Total	35	84,301			

**Table 10:** Mean, standard deviations, and confidence interval of E as a function of angular orientations.

Angular Orientation	N	Mean	StDev	95% CI
0 degrees	9	2508.60	25.20	(2474.69, 2542.50)
30 degrees	9	2483.0	52.7	(2449.1, 2516.9)
60 degrees	9	2479.7	57.8	(2445.8, 2513.6)
90 degrees	9	2489.2	56.8	(2455.3, 2523.1)

**Table 11:** DOE setup for E as a function of build orientation.

Front	Side	Top
2508.57	2553.24	2459.33
2417.25	2565.51	2477.90
2462.01	2537.71	2438.71
2479.51	2489.12	2409.25
2513.16	2490.35	2479.51
2526.14	2449.75	2537.71
2559.82	2508.57	2537.71
2430.41	2479.51	2537.71
2434.02	2479.51	2413.92
2413.92	2479.51	2553.24
2508.57	2417.25	2565.51
2508.57	2462.01	2559.82

**Table 12:** ANOVA results for E as a function of build orientation.

Source	DF	Adj SS	Adj MS	F-Value	*p*-Value
Build orientations	2	1926	963.1	0.39	0.683
Error	33	82,374	2496.2		
Total	35	84,301			

**Table 13:** Mean, standard deviation, and confidence intervals for E as a function of build orientations.

Build Orientations	N	Mean	StDev	95% CI
Front	12	2480.2	47.9	(2450.8, 2509.5)
Side	12	2492.7	42.9	(2463.3, 2522.0)
Top	12	2497.5	57.9	(2468.2, 2526.9)

**Table 14:** DOE setup for UTS as a function of angular orientations.

0 Degrees	30 Degrees	60 Degrees	90 Degrees
53.75	49.09	50.37	52.26
53.75	51.77	52.78	52.50
53.75	53.92	53.50	52.86
52.26	52.94	51.98	50.91
52.26	51.51	51.73	51.87
52.26	52.10	51.05	53.75
50.91	50.60	50.32	52.26
50.91	50.37	49.09	52.86
50.91	51.98	52.94	52.50

**Table 15:** ANOVA results for UTS as a function of angular orientations.

Angular Orientations	DF	Adj SS	Adj MS	F-Value	*p*-Value
Factor	3	5.904	1.968	1.26	0.305
Error	32	49.999	1.562		
Total	35	55.903			

**Table 16:** Mean, standard deviation, and confidence interval for UTS as a function of angular orientations.

Angular Orientations	N	Mean	StDev	95% CI
0 degrees	9	52.307	1.230	(51.458, 53.155)
30 degrees	9	51.587	1.433	(50.738, 52.435)
60 degrees	9	51.529	1.443	(50.680, 52.378)
90 degrees	9	52.419	0.775	(51.570, 53.268)

**Table 17:** DOE setup for UTS as a function of build orientations.

Front	Side	Top
53.75	52.94	51.05
49.09	51.98	52.10
50.37	50.91	50.60
52.26	51.51	50.32
51.77	51.73	52.26
52.78	51.87	50.91
52.50	53.75	50.91
53.92	52.26	50.91
53.50	52.26	52.86
52.86	52.26	52.94
53.75	49.09	51.98
53.75	50.37	52.50

**Table 18:** ANOVA results for UTS as a function of build orientations.

Build Orientations	DF	Adj SS	Adj MS	F-Value	*p*-Value
Factor	2	5.846	2.923	1.93	0.162
Error	33	50.058	1.517		
Total	35	55.903			

**Table 19:** Mean, standard deviation, and confidence interval for UTS as a function of build orientations.

Build Orientations	N	Mean	StDev	95% CI
Front	12	52.525	1.497	(51.802, 53.248)
Side	12	51.744	1.206	(51.021, 52.468)
Top	12	51.612	0.924	(50.888, 52.335)

**Table 20:** DOE setup for percentage elongation as a function of angular orientations.

0 degrees	30 degrees	60 degrees	90 degrees
5.94	4.27	4.82	4.45
5.94	4.94	4.51	4.68
5.94	4.56	4.29	4.77
4.45	4.89	4.32	4.90
4.45	4.88	4.98	4.66
4.45	5.47	4.84	5.94
4.90	4.87	4.70	4.77
4.90	4.82	4.27	4.45
4.90	4.32	4.89	4.68

**Table 21:** ANOVA results for percentage elongation as a function of angular orientations.

Source	DF	Adj SS	Adj MS	F-Value	*p*-Value
Angular orientations	3	1.046	0.3486	1.64	0.199
Error	32	6.792	0.2123		
Total	35	7.838			

**Table 22:** Mean, standard deviation, confidence interval for percentage elongation as a function of angular orientations.

Angular Orientations	N	Mean	StDev	95% CI
0 degrees	9	5.097	0.662	(4.784, 5.409)
30 degrees	9	4.780	0.363	(4.467, 5.093)
60 degrees	9	4.6244	0.2808	(4.3116, 4.9373)
90 degrees	9	4.811	0.448	(4.498, 5.124)

**Table 23:** DOE setup for percentage elongation as a function of build orientations.

Front	Side	Top
5.94	4.89	4.84
4.27	4.32	5.47
4.82	4.90	4.87
4.45	4.88	4.70
4.94	4.98	4.45
4.51	4.66	4.90
4.68	5.94	4.90
4.56	4.45	4.90
4.29	4.45	4.77
4.77	4.45	4.89
5.94	4.27	4.32
5.94	4.82	4.68

**Table 24:** ANOVA results for percentage elongation as a function of build orientations.

Build Orientations	DF	Adj SS	Adj MS	F-Value	*p*-Value
Factor	2	0.1914	0.09568	0.41	0.665
Error	33	7.6466	0.23172		
Total	35	7.8380			

**Table 25:** Mean, standard deviation, and confidence interval for percentage elongation as a function of build orientations.

Build Orientations	N	Mean	StDev	95% CI
Front	12	4.926	0.643	(4.643, 5.209)
Side	12	4.751	0.450	(4.468, 5.034)
Top	12	4.8075	0.2814	(4.5248, 5.0902)

**Table 26:** DOE setup for Ra as a function of build orientations.

Front	Side	Top
1.37	2.47	2.50
2.90	2.90	3.10
3.30	2.23	2.00
1.60	2.30	1.80
2.20	2.00	2.23
2.97	1.90	2.23
1.50	1.60	2.23
3.00	1.60	1.57
2.07	1.60	2.47
1.57	2.90	2.90
1.37	3.30	1.60
1.37	1.37	1.50

**Table 27:** ANOVA results for Ra as a function of build orientations.

Build Orientations	DF	Adj SS	Adj MS	F-Value	*p*-Value
Factor	2	0.0481	0.02406	0.06	0.942
Error	33	13.2221	0.40067		
Total	35	13.2702			

**Table 28:** Mean, standard deviation, confidence interval for Ra as a function of build orientations.

Build Orientations	N	Mean	StDev	95% CI
Front	12	2.102	0.748	(1.730, 2.473)
Side	12	2.181	0.616	(1.809, 2.553)
Top	12	2.177	0.514	(1.806, 2.549)

**Table 29:** DOE setup for Ra as a function of angular orientations.

0 Degrees	30 Degrees	60 Degrees	90 Degrees
1.37	2.90	3.30	1.60
1.37	2.20	2.97	1.50
1.37	3.00	2.07	1.57
1.60	2.47	2.90	2.23
1.60	2.30	2.00	1.90
1.60	3.10	2.50	1.37
2.23	2.00	1.80	1.60
2.23	3.30	2.90	1.57
2.23	2.90	2.47	1.50

**Table 30:** ANOVA results for Ra as a function of angular orientations.

Angular Orientations	DF	Adj SS	Adj MS	F-Value	*p*-Value
Factor	3	7.812	2.6039	15.26	0.000
Error	32	5.459	0.1706		
Total	35	13.270			

**Table 31:** Mean, standard deviation, confidence interval for Ra as a function of angular orientations.

Angular Orientations	N	Mean	StDev	95% CI
0 degrees	9	1.733	0.386	(1.453, 2.014)
30 degrees	9	2.686	0.453	(2.405, 2.966)
60 degrees	9	2.546	0.511	(2.265, 2.826)
90 degrees	9	1.6489	0.2598	(1.3685, 1.9293)
